# Influence of Lignin Content and Pressing Time on Plywood Properties Bonded with Cold-Setting Adhesive Based on Poly (Vinyl Alcohol), Lignin, and Hexamine

**DOI:** 10.3390/polym14102111

**Published:** 2022-05-22

**Authors:** Muhammad Adly Rahandi Lubis, Ahmad Labib, Sudarmanto, Fazhar Akbar, Arif Nuryawan, Petar Antov, Lubos Kristak, Antonios Nikolaos Papadopoulos, Antonio Pizzi

**Affiliations:** 1 Research Center for Biomass and Bioproducts, National Research and Innovation Agency of Indonesia, Cibinong 16911, Indonesia; sudarmantokm46@gmail.com (S.); fazharbio46@yahoo.co.id (F.A.); 2 Research Collaboration Center for Biomass and Biorefinery between BRIN and Universitas Padjadjaran, National Research and Innovation Agency of Indonesia, Cibinong 16911, Indonesia; 3 Department of Forest Products Technology, Faculty of Forestry, Universitas Sumatera Utara, Kwala Bekala Campus, Medan 20355, Indonesia; alabib602@gmail.com; 4 Faculty of Forest Industry, University of Forestry, 1797 Sofia, Bulgaria; p.antov@ltu.bg; 5 Faculty of Wood Sciences and Technology, Technical University in Zvolen, 96001 Zvolen, Slovakia; kristak@tuzvo.sk; 6 Laboratory of Wood Chemistry and Technology, Department of Forestry and Natural Environment, International Hellenic University, GR-661 00 Drama, Greece; 7 LERMAB, Faculte des Sciences, University of Lorraine, Blvd. des Aiguillettes, 54000 Nancy, France; antonio.pizzi@univ-lorraine.fr

**Keywords:** adhesion, bio-based adhesives, cold pressing, cohesion, formaldehyde emission, hexamine, lignin, PVOH, wood-based panels, plywood

## Abstract

The sustainability, performance, and cost of production in the plywood industry depend on wood adhesives and the hot-pressing process. In this study, a cold-setting plywood adhesive was developed based on polyvinyl alcohol (PVOH), high-purity lignin, and hexamine. The influence of lignin content (10%, 15%, and 20%) and cold-pressing time (3, 6, 12, and 24 h) on cohesion, adhesion, and formaldehyde emission of plywood were investigated through physical, chemical, thermal, and mechanical analyses. The increased lignin addition level lowered the solids content, which resulted in reduced average viscosity of the adhesive. As a result, the cohesion strength of the adhesive formulation with 10% lignin addition was greater than those of 15% and 20% lignin content. Markedly, the adhesive formulation containing a 15% lignin addition level exhibited superior thermo-mechanical properties than the blends with 10% and 20% lignin content. This study showed that 10% and 15% lignin content in the adhesive resulted in better cohesion strength than that with 20% lignin content. However, statistical analysis revealed that the addition of 20% lignin in the adhesive and using a cold-pressing time of 24 h could produce plywood that was comparable to the control polyurethane resins, i.e., dry tensile shear strength (TSS) value of 0.95 MPa, modulus of rupture (MOR) ranging from 35.8 MPa, modulus of elasticity (MOE) values varying from 3980 MPa, and close-to-zero formaldehyde emission (FE) of 0.1 mg/L, which meets the strictest emission standards. This study demonstrated the feasibility of fabricating eco-friendly plywood bonded with PVOH–lignin–hexamine-based adhesive using cold pressing as an alternative to conventional plywood.

## 1. Introduction

The sustainability, performance, and cost of production in the plywood industry depend on wood adhesives and the hot-pressing process [1,2]. Plywood is produced dominantly using urea-formaldehyde (UF) resins, with an estimated global consumption of around 30–40 million metric tons in 2020 [2,3]. UF resins are classified as thermosetting aminoplastic resins and the main wood adhesives used in the plywood industry [4,5,6]. The main advantages of UF resins are related to their relatively low cost, ease of handling, fast curing, short pressing time, aqueous solubility, lack of color, and good adhesion strength [7,8]. However, it needs to elevate the press temperature to harden and form crosslinked thermosetting polymers [9,10]. In addition to the need to elevate temperature, UF resins are characterized by deteriorated hydrophobic properties and formaldehyde-based resins that are hazardous and carcinogenic [11,12,13]. The concern with sustainability in manufacturing eco-friendly plywood was more prominent in the 1990s, focusing on the life cycle assessment of adhesive production and the hot-pressing process [14,15,16,17,18,19,20]. A viable option to achieve sustainability in the green manufacturing of plywood is by using bio-based wood adhesives and a cold-pressing process for manufacturing the panels [21,22].

Bio-based wood adhesives have gained tremendous research and industrial interest and have become one of the main research topics in the last decade due to the increased environmental awareness, the need for more sustainable wood-based products, and the stringent legislative requirements related to hazardous formaldehyde emission from wood composites [23]. As one of the bio-sources, lignin is considered to be the second most abundant aromatic polymer in nature, just after cellulose [24,25,26]. It is obtained as a by-product of the paper pulp industry with an estimated annual global production level of 50–75 million tons, of which less than 2% is utilized in value-added applications, while the rest is mostly burned for energy or directly managed as waste [27]. The development of adhesives for manufacturing low-emission wood-based panels is a feasible option for the efficient utilization of technical lignins (lignosulfonates, hydrolysis, organosolv, ionic, or kraft lignin) [28,29,30,31,32,33,34,35]. It should be noted that most lignin-based wood adhesives are tested mainly in laboratory conditions and still have not been introduced into industrial practice. The simplest way to use lignin in the form of wood-based panel adhesives is based on the partial substitution of phenol in phenolic resins [30,32,35]. Although the phenolic structure of lignin provides high hydrophobicity and low dispersion properties for the development of bio-based adhesives, its aromatic structure decreases its reactivity, so the development of lignin-based adhesives requires previous stages of functionalization of the lignin structure through phenolic and aliphatic groups as well as aromatic rings [30,32,35].

Another alternative to formaldehyde in the production of wood adhesives is hexamethylenetetramine or hexamine. The dominant use of hexamine is in the production of phenolic resin moulding compounds, where it is added as a hardening component [29,35]. Glyoxalated wheat straw lignin mixed with mimosa tannin and hexamine as a hardener was used as a wood adhesive for interior particleboard panels with a reduced formaldehyde emission with proportions of 40–60% and 50–50% for lignin and tannin, respectively [36]. Another study showed that softwood kraft lignin was glyoxylated and formulated with tannin-hexamine [35]. The lignin-based adhesive was prepared with a 60% tannin–40% lignin proportion. The glyoxalated lignin–tannin–hexamine-based adhesive can be used for bonding plywood. The curing enthalpy for the lignin-based adhesive with hexamine as hardener is usually higher than glyoxal and formaldehyde. The highest rate of chemical cure was achieved using paraformaldehyde as hardener followed by glyoxal and hexamine, which required higher curing temperatures to achieve a complete chemical cure. Furthermore, the rate of increase of adhesion strength decreased in the order hexamine > glyoxal > formaldehyde, where the rigidity after curing increased in the same order [35].

Given the rapid development of engineered wood products, the requirement for cold-setting adhesive for on-site bonding is growing quickly. Epoxies, polyurethane (PU) resins, and resorcinol-formaldehyde are common types of adhesives used for on-site bonding [37,38]. However, they are derived from unsustainable fossil-based constituents which are not renewable. Among them, PU resin adhesive is extensively used in the wood industry because of its superior performance, such as strong adhesion, good thermal and chemical stability, and required low curing temperature [39]. Plywood bonded with PU resins showed dry tensile shear strength (TSS) in the range of 1.5–2.5 MPa, [40,41,42], which is greater than the minimum requirement of 0.7 MPa [43].

By contrast, poly(vinyl alcohol) (PVOH)-based adhesive showed dry TSS values in the range of 0.1–1.2 MPa [44]. The PVOH-based adhesives rely on hydrogen bondings with wood, which have lower bonding strength than the covalent bonds [45]. Therefore, the development of bio-based cold-setting adhesives is necessary. As mentioned above, hexamine is linked to lignin units both by covalent and ionic bonding [35]. To enhance the link of lignin with hexamine, PVOH is used to promote more hydroxy (−OH) groups in the system. PVOH, a highly polar and water-soluble polymer, is well utilized in polymer blends with natural polymeric materials such as cellulose, starch, chitosan, and lignin [46,47,48]. In these systems, good material performance was obtained and attributed to the formation of intermolecular hydrogen bonds between the hydroxyl groups of the respective polymers. It is expected that the blending of PVOH, lignin, and hexamine could provide an alternative cold-setting wood adhesive.

Therefore, this study aimed to develop an alternative cold-setting wood adhesive for plywood based on PVOH, lignin, and hexamine and to investigate the influence of lignin content on the cohesion, adhesion strength, and formaldehyde emission of plywood. The analyses used included functional group analysis using Fourier transform infrared spectroscopy (FTIR), thermal analysis using differential scanning calorimetry (DSC), thermo-mechanical analysis using dynamic mechanical analyzer (DMA), cohesion and flow behavior using a rotational rheometer, adhesion strength using a universal testing machine (UTM), and formaldehyde emission using the desiccator method.

## 2. Materials and Methods 

### 2.1. Materials

Black liquor obtained from the kraft pulping process of *Acacia mangium* was provided by Tanjung Enim Lestari Pulp and Paper Company, Muara Enim, South Sumatra, Indonesia. Chemicals used for isolation and characterization of lignin were hydrochloric acid (HCl 37%, analytical grade, Merck, Darmstadt, Germany), sulfuric acid (H_2_SO_4_ 95–97%, analytical grade, Merck, Darmstadt, Germany), dioxane (1,4-dioxane, analytical grade, Merck, Darmstadt, Germany), acetone (analytical grade, Merck, Darmstadt, Germany), and distilled water. The technical grade of PVOH (±22.000 g/mole), hexamine, and calcium carbonate (CaCO_3_) was purchased from PT. Bratachem (Bogor, Indonesia). As a control adhesive, PU resins (MDI:Polyol 1.2:1, solids content 96.52%, dynamic viscosity 2056.5 mPa·s, and gelation time 187.5 min) was purchased from Anugerah Raya Kencana Company, Tangerang, Indonesia. Sengon (*Paraserianthes falcataria* (L.) Nielsen) wood veneers with a size of 250 mm × 250 mm × 2 mm, moisture content of 6 ± 1%, and without noticeable defects were purchased from the local market (Cibinong, Indonesia) to prepare three-layer plywood panels.

### 2.2. Isolation of Lignin

Lignin was isolated via an acid precipitation method [49]. Approximately 200 g of black liquor and 2000 mL of distilled water were stuffed into a plastic jar. HCl 1 M was added while stirring to adjust the pH of the black liquor from around 12 to 2. The solution was kept for 24 h at room temperature (25 °C) to separate the filtrate and residue. Further, the decantation process was carried out three times, and then the remaining sludge was refrigerated for 24 h. The precipitate was filtered with a Buchner funnel and kept in an oven for 24 h at 45 ℃. Then, the precipitate was powdered using a mortar and filtered by a sieve with an opening size of 40 mesh and retained at size of 60 mesh.

### 2.3. Characterization of Lignin

Basic properties of lignin such as yield, moisture content (MC), ash content, and purity were characterized using various analytical methods. The yield of lignin was calculated by dividing the weight of isolated lignin and black liquor. The MC of lignin was determined by drying lignin samples in an oven at 105 °C for 24 h.

The ash content of lignin was determined by heating the sample in a furnace for 6 h at a temperature of 525 ± 25 °C. Ash content of lignin is calculated according to the following equation [50].

(1) $$Ash\;content\;\left(\%\right)=\frac{\left(C-A\right)}{B}\times 100\%$$

Description:

*A* = weight of empty porcelain cup (g).

*B* = weight of lignin sample (g).

*C* = weight of the oven-dried sample and porcelain cup (g).

The purity of isolated lignin was measured according to the standard procedure [51,52]. Approximately 0.3 g of lignin were added to a vial bottle. After that, 3 mL of H_2_SO_4_ 72% were added and mixed with a magnetic stirrer at 150 rpm for 2 h. A blank solution was prepared by adding 3 mL of H_2_SO_4_ 72% and 84 mL of distilled water. Furthermore, the sample was heated by autoclave for 1 h at a temperature of 121 °C. The solution was filtered using an IG3 filter glass (CTE33, IWAKI, Shizuoka, Japan). The residue (acid-insoluble lignin) was put in the oven at 105 °C for 24 h. The filtrate was diluted in a test tube with a blank solution of 13 times dilution. The solution was stirred with a vortex, and its absorbance was measured using a UV-Vis spectrophotometer (UV-1800, Shimadzu, Kyoto, Japan) at a wavelength of 240 nm. Acid soluble lignin (ASL) and acid-insoluble lignin (AIL) were calculated using the following equations [51,52].

(2) $$ASL\;\left(\%\right)=\frac{UVabs\times volume\;filtrate\times dilution}{\varepsilon \times A\times cuvette\;length}\times 100\%$$

(3) AIL (%)=AIR(%)−Ash content (%)

(4) $$Acid\;Insoluble\;Residue\;\left(AIR\right)\;\left(\%\right)=\frac{C-B}{A}\times 100\%$$

Description:

*ε* = absorptivity constant of biomass at a specific wavelength (L/g·cm)

*A* = weight of sample without moisture content (g).

*B* = dry weight of IG3 filter glass (g).

*C* = dry weight of IG3 filter glass and AIL (g).

Functional groups of isolated lignin were investigated using Fourier Transform Infra-Red (FTIR) spectroscopy (SpectrumTwo, PerkinElmer Inc., Hopkinton, MA, USA) with the Universal Attenuated Total Reflectance (UATR) method. The average accumulation was recorded as 16 scans at a resolution of 4 cm^−1^ at wavelengths ranging from 4000–400 cm^−1^ at 23 ± 2 °C. Each spectrum was normalized using min–max normalization in Spectrum software (Ver. 10.5.3, Perkin Elmer Inc., Hopkinton, MA, USA).

The thermal properties of isolated lignin were analyzed using Differential Scanning Calorimetry (DSC4000, Perkin Elmer Inc., Hopkinton, MA, USA). Around 4 mg of sample were weighed into a standard aluminum pan (40 μL). The samples were heated in a nitrogen atmosphere with a flow rate of 20 mL/min and temperatures ranging from −30 to 180 °C with a heating rate of 10 °C/min. The peak temperature (*Tp*) values were calculated automatically using the pyris 11 software (Version 11.1.1.0492, Pyris, Washington, MA, USA).

### 2.4. Preparation of PVOH–Lignin–Hexamine-Based Adhesive

Prior to the blending process, lignin solution was prepared by mixing lignin in acetone at a ratio of 1:10, according to the preliminary research. Furthermore, PVOH beads were dissolved in distilled water at 80 °C to obtain a PVOH solution of 15% *w*/*v* [53,54], and hexamine 15% *w/v* was prepared by dissolved hexamine powder in distilled water at 27 ± 2 °C. The mixing process of adhesive was conducted at a temperature of 27 ± 2 °C under a stirring rate of 300 rpm according to the formulation in Table 1. As a control, PU resin adhesive was used as a commercial cold-setting adhesive.

### 2.5. Characterization of Adhesive

The non-volatile solids content of PVOH–lignin–hexamine-based adhesives at different lignin contents was determined by drying the samples in an oven at 105 ± 2 °C for 3 h and dividing the dry samples with wet samples. A rotational rheometer (Rheolab QC, Anton Paar, Graz, Austria) was used to determine the viscosity of PVOH–lignin–hexamine- based adhesives. The viscosity of PVOH–lignin–hexamine-based adhesives was measured using spindle No. 27 at a constant speed of 100 rpm and at 27 ± 2 °C. A gel time meter (Techne GT-6, Coleparmer, Vernon Hills, IL, USA) was utilized to measure the gel time of PVOH/lignin/hexamine based adhesive at different lignin contents at 27 ± 2 °C. All measurements were conducted in triplicate.

Rheological properties of PVOH–lignin–hexamine-based adhesives at different lignin contents were investigated using a rotational rheometer (RheolabQC, AntonPaar, Graz, Austria). Shear stress, shear strain, dynamic viscosity, torque, and cohesion strength of the adhesive were evaluated at a temperature of 25 ± 1 °C under a dynamic shear rate of 1–500/s.

A FTIR spectrometer coupled with UATR (Spectrum Two, Perkin Elmer Inc., Hopkinton, MA, USA) was used to investigate the functional groups of PVOH–lignin–hexamine-based adhesive at different lignin contents in the range of 400–4000 cm^−1^ with a resolution of 2 cm^−1^ and 16 scans per sample. A min–max normalization was carried out to normalize the spectrum of adhesive (Ver. 10.5.3, Perkin Elmer Inc., Hopkinton, MA, USA).

A DSC (DSC 4000, Perkin Elmer, Hopkinton, MA, USA) was utilized to analyze the thermal properties of PVOH–lignin–hexamine-based adhesives. Around 5.0 ± 0.2 mg of sample were scanned from −30 °C to 90 °C at a 10 °C/min heating rate. The nitrogen gas was purged with 20 mL/min of flow rate. The peak temperature (*Tp*) was calculated using Pyris Software (Ver. 11.1.1.0492, Pyris, Washington, MA, USA).

DMA (DMA 8000, Perkin Elmer Inc., Hopkinton, MA, USA) was performed to investigate the thermo-mechanical properties of PVOH–lignin–hexamine-based adhesive at different lignin contents. Each adhesive was used to bond two Whatman filter papers, with a glue spread of 180 g/m^2^, to prepare a specimen with the dimensions of 50 mm × 8 mm × 0.2 mm. All specimens were precured in an oven at 50 °C for 5 min before the DMA analysis. The storage modulus (*E′*), loss modulus (*E″*), and tan delta of each specimen were determined at a frequency of 1 Hz, strain level of 0.01%, and heating rate of 2 °C/min in the scanning range of 20–60 °C in the dual cantilever mode.

### 2.6. Fabrication of Plywood Bonded with PVOH–Lignin–Hexamine-Based Adhesive

Plywood samples were fabricated using Sengon wood veneers bonded with PVOH–lignin–hexamine-based adhesive at different lignin contents. Approximately 15% of CaCO_3_ was added based on the solids content of the adhesive as a filler. A total of 39 samples of three-layer plywood with a target size of 300 mm × 300 mm × 6 mm were fabricated by spreading the glue mixture on the veneer surface with a glue spread of 180 g/m^2^ using the double spread method (Table 2). Plywood was pressed at 27 ± 2 °C under an average specific pressing pressure of 5.0 MPa for 3, 6, 12, and 24 h. All samples were then conditioned at 20 °C for two weeks prior to the plywood characterization. As a control, plywood bonded with PU adhesive was also prepared at a glue spread of 180 g/m^2^ using the double spread method and cold-pressed at 27 ± 2 °C under an average specific pressing pressure of 5.0 MPa for 3 h.

### 2.7. Examination of Plywood Properties

**Figure 1:**
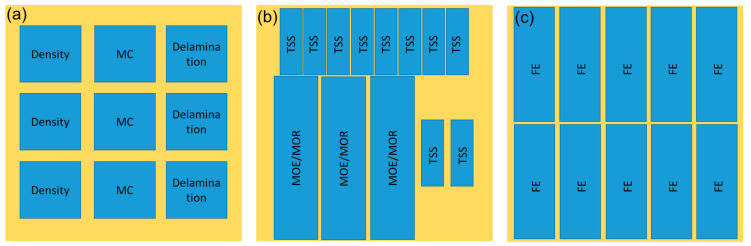
The cutting plan of the test specimens of plywood. (**a**) Physical test sample, (**b**) mechanical test sample, (**c**) FE test sample.

The properties of the plywood were evaluated according to Japanese Agriculture Standard (JAS) No. 233:2003 [43], and the cutting plan of the test specimens is displayed in Figure 1. Plywood samples with the dimensions of 75 mm × 75 mm × 6 mm were used for density, MC, and delamination measurement. Briefly, the density of plywood was determined by comparing the mass and volume of plywood, while the MC was obtained by dividing the initial and final mass after drying at 103 ± 2 °C for 24 h. Meanwhile, the delamination test was determined according to Type II plywood for general use [43]. Plywood samples were immersed in hot water for 2 h and then dried at 60 ± 3 °C for 3 h. The delamination percentage was obtained by dividing the total length of delaminated lines and the total length of glue lines. The length of the non-delaminated portion on the same bonding layer of the sample shall be not less than 2/3 of the length of each side (>50 mm).

The tensile shear strength (TSS), modulus of rupture (MOR), modulus of elasticity (MOE), and formaldehyde emission (FE) measurements of plywood samples were carried out according to JAS No. 233:2003 [43]. The TSS values of 10 specimens with the dimensions of 80 mm × 25 mm × 6 mm, were measured using a universal testing machine (UTM AGX Series, Shimadzu, Kyoto, Japan) at a crosshead speed of 2 mm/min using a load cell of 10 kN. Wood failure analysis was performed using the TSS samples right after the analysis. The FE values were determined by placing 10 plywood samples (150 mm × 50 mm × 6 mm) together with 300 mL of distilled water in a desiccator at 20 ± 2 °C for 24 h. Approximately 25 mL of the solution was pipetted into 100-mL flasks and mixed with 25 mL acetyl acetone–acetic acid ammonium solutions. The mixtures were heated for 10 min at 65 ± 2 °C in a water bath and then cooled to room temperature. The FE was measured using UV–visible spectrophotometry (UV-1800, Shimadzu, Kyoto, Japan) at a wavelength of 412 nm.

### 2.8. Statistical Analysis

The mean values of the mechanical properties of plywood were compared using analysis of variance (ANOVA), and the Duncan multiple range test at α = 0.05 was performed to determine the optimum lignin content and pressing time for plywood. Statistical analysis was conducted using SPSS 21 software (SPSS Inc., Chicago, IL, USA).

## 3. Results and Discussion

### 3.1. Characteristics of Lignin

The characteristics of black liquor and isolated lignin are shown in Table 3. The solids content of black liquor used in this study was around 76.8%. The yield of isolated lignin obtained was 35.9%, and it was lower than the one-stage lignin precipitation of 45.8% [49,55]. This study showed that around 35.9 g of lignin could be isolated from 100 g of black liquor. The yield of isolated lignin is affected by the solids content and lignin content in black liquor from the kraft pulping process as well as the precipitation process to isolate the lignin.

The MC of isolated lignin was 5.1%. This value is relatively low compared to the published results, varying from 5% to 8% [49,55]. Furthermore, the low ash content of 0.3% indicated that the three-stage decantation process to obtain the lignin in this study produced fewer impurities compared to other studies that used a one-stage decantation process with 8.3–19.2% of ash content [49,55]. The ash content is the residual content of combustion of inorganic materials found in lignin.

The determination of lignin purity is based on the AIL and ASL. As presented in Table 3, the AIL content was 82.5%, and ASL content was 12.8%. The high AIL and ASL values determined can be attributed to the three-stage decantation process during isolation. The decantation process involved washing with distilled water to remove impurities and other substances, thus increasing the total lignin generated from the isolation process. The level of purity of the isolated lignin was relatively high, namely 95.3%. This result is higher than the published works that used similar sources of black liquor and acid precipitation methods [49,55], calculating around 60.4% of lignin purity. The difference in the level of lignin purity is influenced by differences in mineral content in the lignin.

### 3.2. Properties of Adhesive

**Figure 2:**
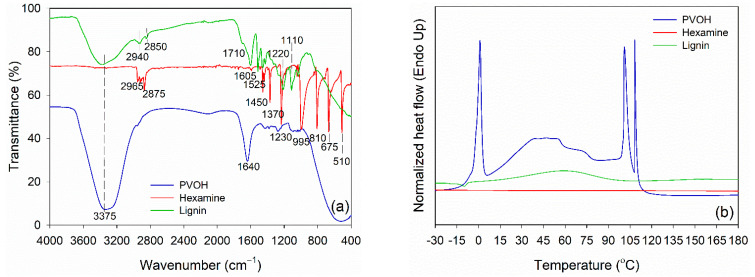
Characteristics of PVOH, hexamine, and lignin. (**a**) FTIR spectra, (**b**) DSC thermograms.

Prior to blending, functional groups of PVOH, lignin, and hexamine were analyzed using FTIR spectroscopy, and the result is displayed in Figure 2a. PVOH and lignin had a typical hydroxy group (−OH) at 3375 cm^−1^. Intermolecular hydrogen bonds of PVOH were observed at 1640 cm^−1^. The −OH group is an important functional group for linking with the −NH_2_ group of hexamine at wavenumber 3400 cm^−1^ and 1450 cm^−^^1^. Various vibrations of methylene (−CH_2_−) of hexamine were detected at wavenumber 2965 cm^−1^, 2875 cm^−1^, 1450 cm^−1^, 1370 cm^−1^, 1230 cm^−1^, 995 cm^−1^, 810 cm^−1^, 675 cm^−1^, and 510 cm^−1^. The methylene and methoxyl groups of lignin were observed at wavenumber 2940 cm^−1^ and 2850 cm^−1^, respectively. Furthermore, the C=O stretching of lignin was detected in the unconjugated ketone and aldehyde group at wavenumbers 1710 cm^−1^. The aromatic C=C stretching of the aromatic ring of lignin has a wavenumber of 1605 cm^−1^, and C=C of aromatic skeletal vibrations of the phenyl-propane (C_9_) skeleton has a wavenumber of 1525 cm^−1^ [58,59]. The C–O–C of the guaiacyl ring and C–O–C of the aromatic acetyl groups were defined in the wavenumber 1220 cm^−1^. The wavenumber 1110 cm^−1^ indicated the existence of the aromatic C-H deformation of syringol [59,60].

DSC was used to evaluate the thermal properties of PVOH, lignin, and hexamine (Figure 2b). The endothermic peak temperature of PVOH was observed at around 0.0 °C, which was attributed to the melting temperature (T_m_) of water in PVOH. This result is in accordance with the published work that showed the T_m_ of PVOH was in the range of 0.0 to 1.0 °C [61]. The endothermic peak of lignin at a temperature around 60 °C was observed due to the evaporation of moisture in the lignin. This peak is usually found at temperatures below 100 °C, specifically at 56–65 °C for lignin [49]. Furthermore, the glass transition temperature (T_g_) occurs whenever the lignin structure changes, potentially reducing lignin’s rigidity. Because of its complex structure, the T_g_ value of lignin is sometimes challenging to detect. However, the range of variations in the curve can occasionally be seen [62]. The T_g_ value was detected at a temperature around 151 °C, which was generally between 100–180 °C [59,60]. Because of the wide range of T_g_, there are variances in the flexibility and stiffness of lignin at high temperatures, which will influence its use as a wood adhesive. Meanwhile, no endothermic peak was detected in hexamine at a range of −30 °C to 180 °C, showing no reaction occurred in a neat solution of hexamine under an N_2_ atmosphere.

The main properties of PVOH–lignin–hexamine-based adhesives at different lignin contents are summarized in Table 4. In general, the solids content and viscosity of PVOH–lignin–hexamine-based adhesives decreased with higher lignin content. This indicated that the incorporation of lignin lowered the solids content due to the presence of acetone in the lignin solution. As a result, the viscosity decreased following the solids content of the adhesive. In this study, the base of adhesive, PVOH 15% *w*/*v*, had an average dynamic viscosity of around 2000 mPa·s. After the addition of lignin, the average viscosity decreased to 1894.6 mPa·s for 10% lignin content and further lowered to 968.5 mPa·s with 20% lignin addition. By contrast, the control adhesive, PU resins, had high solids content and viscosity of 96.52% and 2056.5 mPa·s, respectively. The result showed that the gelation time of PVOH–lignin–hexamine-based adhesives at different lignin contents could not be detected by the gel time meter, meaning that the gelation process of the adhesive over the limit of the instrument, which was 999 min or around 17 h. By contrast, PU resin adhesive showed a gel time value of 187.5 min or around 3 h.

**Figure 3:**
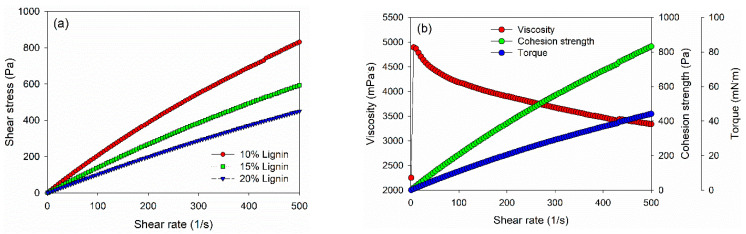

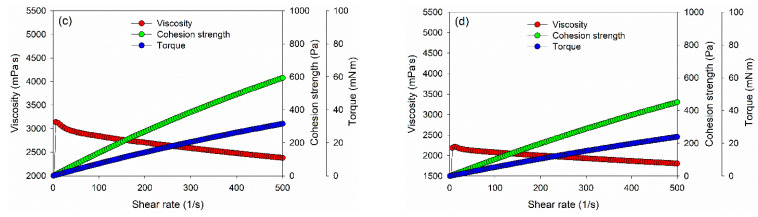
Viscosity, torque, and cohesion strength of PVOH–lignin–hexamine blends with different contents of lignin: (**a**) shear rate–shear stress curve, (**b**) 10% lignin, (**c**) 15% lignin, (**d**) 20% lignin.

Rheological properties of PVOH–lignin–hexamine-based adhesives at different lignin contents were investigated using a rotational rheometer (Figure 3). The shear stress value increased as a function of the shear rate. The 10% addition of lignin in the adhesive gave a higher shear stress than those of 15% and 20% of lignin content (Figure 3a). As a result, PVOH–hexamine with 10% lignin content had an initial dynamic viscosity of around 2250 mPa·s at a shear rate of 1/s, then the value increased to 4800 mPa·s at a shear rate of 10/s, and finally, the value decreased to around 3300 mPa·s at a shear rate of 500/s (Figure 3b). The cohesion strength of PVOH–hexamine with10% lignin content increased from 16 Pa at a shear rate of 1/s to 831 Pa at a shear rate of 500/s. A similar trend was also observed for the torque value of the adhesive.

However, the dynamic viscosity, cohesion strength, and torque values of PVOH–lignin–hexamine-based adhesives decreased with the addition of 15% and 20% lignin (Figure 3c,d). The result showed that the initial dynamic viscosity of adhesive at 15% and 20% addition of lignin was around 1340 mPa·s and 625 mPa·s at a shear rate of 1/s, respectively, then the value increased to 3100 mPa·s and 2200 mPa·s at a shear rate of 10/s, and finally the dynamic viscosity decreased to 2400 mPa·s and 1800 mPa·s at a shear rate of 500/s. Furthermore, the cohesion strength also decreased compared to the 10% lignin content by 40–50% with the addition of 15% and 20% of lignin, to around 593 Pa and 450 Pa, respectively. A similar trend was also observed for the torque value of the adhesive. The results implied that increasing the lignin content reduced the cohesion strength of the PVOH–lignin–hexamine-based adhesives. This result was supported by solids content and average viscosity of adhesive, which lowered with higher addition of lignin (Table 4).

**Figure 4:**
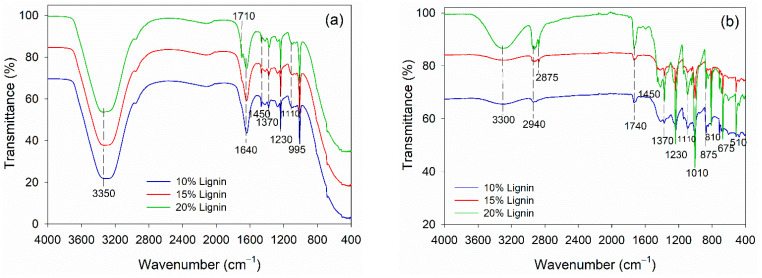
FTIR spectra of PVOH–lignin–hexamine blends with different lignin contents: (**a**) liquid adhesive, (**b**) cured adhesive.

The alteration in functional groups of PVOH–lignin–hexamine-based adhesives at different lignin contents before and after curing was observed using FTIR spectroscopy (Figure 4). The adhesives were based on PVOH; therefore, the liquid adhesive had a typical peak of free −OH group at 3350 cm^−1^ and intermolecular hydrogen bonds at 1640 cm^−1^ (Figure 4a). The addition of lignin did not alter the functional groups remarkably. The typical C=O stretching of lignin and aromatic C-H deformation of syringol was detected at wavenumbers 1710 cm^−1^ and 1100 cm^−1^, respectively [59,60]. The rest of the functional groups in liquid adhesive belonged to the methylene vibrations of hexamine at 1450 cm^−1^, 1370 cm^−1^, 1230 cm^−1^, and 995 cm^−1^. The functional groups of PVOH–lignin–hexamine-based adhesives altered remarkably after curing the adhesive in an oven at 105 ± 3 °C for 3 h (Figure 4b). The wavenumber at 3300 showed a peak secondary amine resulting from the reaction of PVOH–lignin with hexamine. Hexamine is known for its capability of covalent bonding with the −OH of lignin [30]. In the PVOH–lignin blends, the amount of the −OH group was much greater compared to the lignin alone. Therefore, the blends could enhance the cohesion and adhesion strength of the adhesive. After curing, the vibration of methylene linkages from hexamine was detected at wavenumber 2940 cm^−1^ and 2875 cm^−1^. The wavenumber 1740 cm^−1^ was observed as the C=O group of aldehyde resulting from the decomposition of hexamine at high temperatures. Various vibrations of methylene of hexamine were detected at wavenumber, 1450 cm^−1^, 1370 cm^−1^, 1230 cm^−1^, 810 cm^−1^, 675 cm^−1^, and 510 cm^−1^.

**Figure 5:**
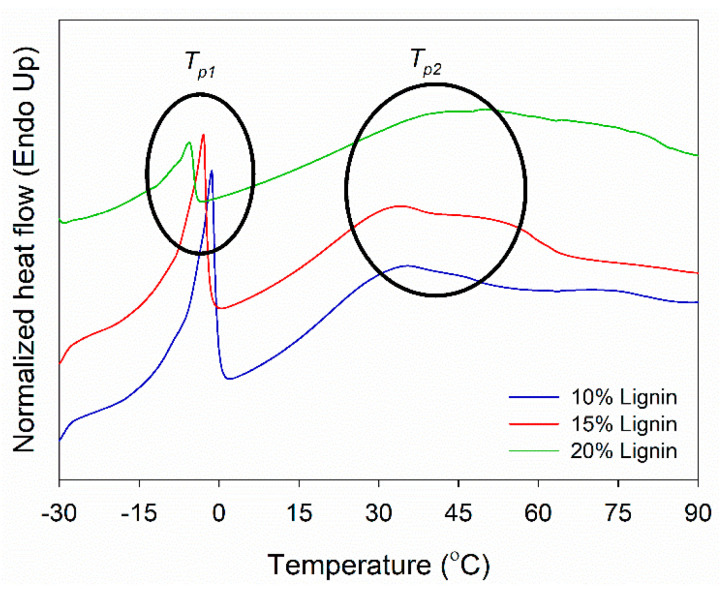
DSC thermograms of PVOH–lignin–hexamine blends at different contents of lignin.

DSC analysis of PVOH–lignin–hexamine-based adhesives at different lignin contents was performed to investigate its thermal curing behavior (Figure 5). DSC measures the heat flow to or from the sample as a function of temperature or time. The T_p1_ showed the melting temperature of water in the adhesive system in the range of −5 to −1 °C. Above this temperature, the adhesive is in the liquid phase. The T_p1_ value decreased with increasing the lignin content. This implied that the addition of lignin lowered the solids content of the adhesive, thus increasing the amount of water and eventually decreasing the T_p1_ value. As supported by the results of FTIR, lignin contained a great amount of hydroxy (−OH) groups at a wavenumber of 3350–3375 cm^−1^, which could increase the amount of water and eventually decrease the T_p1_ value (Figure 2a and Figure 4a,b). The T_p2_ revealed a hardening temperature that the adhesive changed the phase from liquid to gel and finally became solid at 34–48 °C. The T_p2_ value increased by adding a higher amount of lignin into the adhesive. This indicated that the addition of lignin increased the solid’s content of the adhesive, increased the amount of water, resulted in a higher T_g_ value, and eventually increased the T_p2_ value.

**Figure 6:**
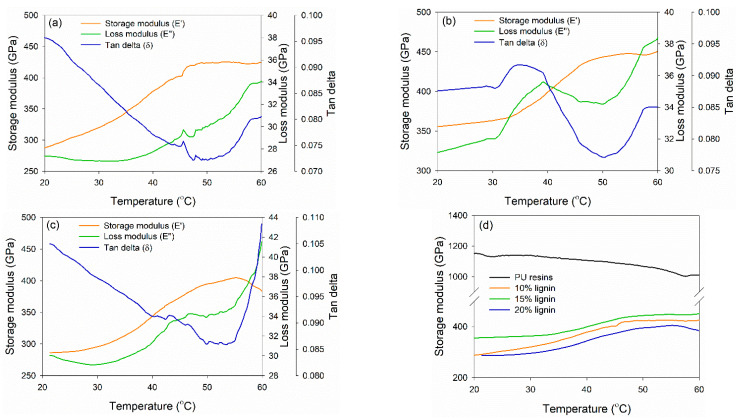
Dynamic mechanical analysis of PVOH–lignin–hexamine blends at different contents of lignin: (**a**) 10% lignin, (**b**) 15% lignin, (**c**) 20% lignin, (**d**) storage modulus of control PU resins and PVOH–lignin–hexamine-based adhesives.

DMA was conducted to investigate the thermo-mechanical properties of PVOH–lignin–hexamine-based adhesives at different lignin contents (Figure 6). DMA measures the mechanical response of viscoelastic materials exposed to the oscillation at various temperatures. The storage modulus (E′), loss modulus (E″), and tan delta (the ratio of E″ to E′) of each adhesive were compared. E′ is a measure of the stored energy of the material and depends on the polymer type, temperature, and frequency of oscillation, whereas E″ measures the dissipated energy of a specimen due to the molecular friction occurring in the viscous flow [63]. The PVOH–hexamine with10% lignin had a minimum E′ and maximum E′ of 280 GPa at 20 °C and 425 GPa at 48 °C (Figure 6a). The E′ initially decreased to a minimum and then reached a maximum. The initial decrease of E′ could be due to the softening of adhesive as the temperature increases. After that, E′ started to increase toward a maximum. This was possibly due to the gelation of the adhesive, during which an infinite molecular network began to be formed. The trend of E″ of the adhesive was similar to those of E′. An initial decrease of E″ could be due to the softening of adhesive as the rigidity decreased. The E″ started to increase after reaching the minimum. This result also reflected the gelation of the adhesive, as indicated by tan delta, in which the reaction started to form a network and resulted in efficient energy dissipation. Increasing the content of lignin to 15% also enhanced the E′_max_ value to around 450 GPa at 48 °C, but the E′_max_ decreased at 20% lignin content to around 405 GPa at 56 °C (Figure 6b,c). The trend of E′_min_, E″_min_, E′_max_, and tan delta of 15% and 20% addition of lignin was similar to the E′_max_ value. Figure 6d shows that 15% lignin content resulted in higher E′_max_; however, the value was two times lower than that of PU resin adhesive, which reached 1100 GPa of E′_max_.

### 3.3. Performance of Plywood

The physical and mechanical performance of plywood bonded with PVOH–lignin–hexamine blends at different contents of lignin and different pressing times were evaluated according to JAS No. 233:2003 [43]. Table 5 present the selected physical properties of plywood at different lignin contents and at different pressing times. The average density of plywood bonded with PVOH–lignin–hexamine and control PU resins was 0.42 g/cm^3^. The result showed that adding higher lignin content and increasing the pressing time did not affect the density of plywood. As can be seen in Table 5, higher lignin content increased the MC of plywood. The increased MC values could be due to the presence of hydroxy groups of PVOH–lignin. By contrast, the MC of plywood control was lower than those of plywood bonded with PVOH–lignin–hexamine-based adhesive. All MC values have met the requirement of JAS No. 233:2003, in which the maximum value is 14%. However, the MC values were still higher than commercial plywood bonded with UF resins and manufactured at elevated temperatures [64]. Delamination is an important characteristic of plywood, especially when it is intended for outdoor applications, i.e., exposed to cyclic changes of MC. These changes lead to the formation of mechanical stresses in the glue joint, resulting from the differences in shrinking and swelling of adjacent veneer layers due to the varied MC. The PVOH–lignin–hexamine-based adhesives used in this work exhibited poor wet bonding strength, with a delamination value of 100%. By contrast, plywood control showed 0% of delamination, showing that PU resins had superior wet bonding strength. The delamination of plywood bonded with PVOH–lignin–hexamine did not meet the minimum requirement of JAS No. 233 with a minimum of 50 mm non-delaminated portion or lower than 33.3%. This represents a serious drawback for the industrial application of the tested PVOH–lignin–hexamine-based adhesives compared to the PU wood adhesives. The chemical modification of veneers is a viable approach to increase the dimensional stability of plywood in order to reduce its delamination and deformation [65].

**Figure 7:**
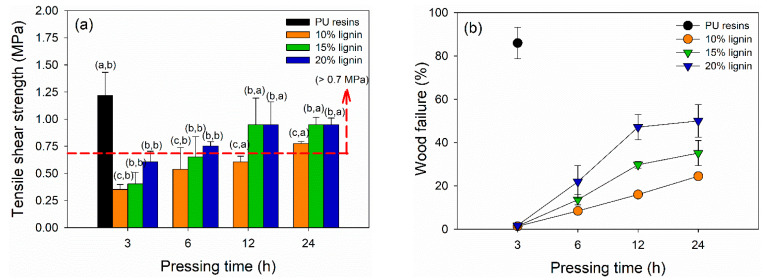
Influence of different lignin contents on the shear strength of plywood at different pressing times: (**a**) tensile shear strength (TSS), (**b**) wood failure.

The dry TSS and wood failure of plywood bonded with PVOH–lignin–hexamine-based adhesives are displayed in Figure 7a,b. The TSS values of plywood increased by adding higher lignin content and longer pressing time. The lowest TSS value of 0.35 MPa was obtained at plywood bonded with PVOH–hexamine and10% lignin after cold pressing for 3 h. The highest one was the plywood bonded with 15% and 20% after cold pressing for 12 h and 24 h, with a similar TSS value of 0.95 MPa. However, the TSS values of plywood bonded with PVOH–lignin–hexamine-based adhesives after cold pressing for 12 h and 24 h were lower than the control, with 1.22 MPa of TSS. This shows that PU resins were superior in adhesion strength even only with the cold pressing of 3 h. Plywood bonded with PVOH–hexamine and15% and -20% lignin after cold pressing for 12 h and 24 h met the minimum requirement of 0.7 MPa [43]. Table 6 summarize the statistical analysis of mechanical properties of plywood bonded with PVOH–lignin–hexamine-based adhesive at different lignin contents and pressing times. Lignin content, pressing time, and their interaction showed a significant influence on the TSS values of plywood. The *p*-value revealed that lignin content and interaction of lignin content and pressing time had a more significant influence on the TSS values compared with the pressing time alone. Statistical analysis suggested that a combination of PVOH–hexamine with 20% lignin adhesive and cold pressing for 24 h could produce plywood with comparable TSS value to the control PU resins. Several studies reported that plywood bonded with UF or melamine–urea–formaldehyde resins using hot pressing for 4–10 min had TSS values ranging from 1.0 to 1.4 MPa [7,12,66,67,68,69,70]. Markedly, a chemical modification of lignin is needed to increase its reactivity, aimed at shortening the cold-pressing time, improving the bonding strength of plywood, and increasing the production capacities for manufacturing plywood panels by cold pressing.

The result of wood failure also showed a similar trend with the TSS, indicating that higher lignin content and longer pressing time enhanced the adhesion strength and increased the wood failure. Plywood prepared with 3 h of cold pressing had wood failure ranging from 1.3–1.8%, showing that the type of failure was a cohesive failure. In this type, the PVOH–lignin–hexamine-based adhesive could not hold the bonding integrity owing to the short cold pressing time and low cohesive strength. By contrast, control plywood had a wood failure of around 86%, indicating the PU resins had superior cohesive strength to maintain the bonding integrity with only 3 h of cold pressing.

**Figure 8:**
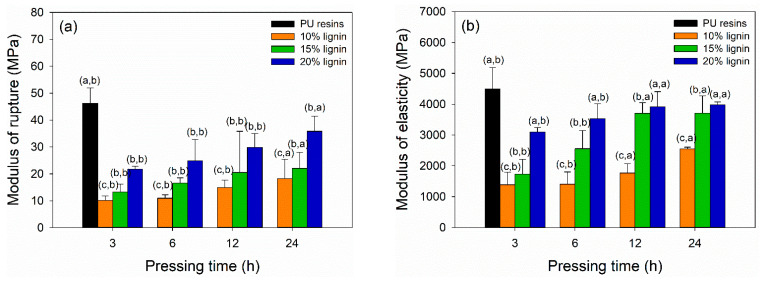
Effect of different lignin contents on bending strength of plywood at different pressing times: (**a**) modulus of rupture, (**b**) modulus of elasticity.

The bending strength of plywood bonded with PVOH–lignin–hexamine blends at different lignin contents and prepared for different pressing times are displayed in Figure 8a,b. The MOR and MOE values of plywood increased by adding higher lignin content and longer pressing time. The highest MOR and MOE values were obtained for plywood bonded with PVOH–hexamine and 20% lignin after cold pressing for 24 h, with a value around 36 MPa and 4000 MPa, respectively. By contrast, control plywood bonded with PU resins showed superior MOR and MOE values of 46 MPa and 4500 MPa, respectively, after cold pressing for just 3 h. The result showed that the MOR and MOE values of plywood bonded with PVOH–hexamine and 20% lignin after cold pressing for 24 h had a comparable value with control plywood. Additional modification of lignin to increase lignin’s reactivity is needed to shorten the cold-pressing time. Table 6 summarizes the statistical analysis of mechanical properties of plywood bonded with PVOH–lignin–hexamine-based adhesive at different lignin contents and pressing times. Lignin content, pressing time, and their interaction showed a significant influence on the MOR and MOE values of plywood. The *p*-value revealed that lignin content and interaction of lignin content and pressing time had a more significant influence on the MOR and MOE values compared with the pressing time alone. Statistical analysis suggested that a combination of PVOH–hexamine with 20% lignin adhesive and cold pressing for 24 h could produce plywood with comparable MOR and MOE values to the control PU resins.

**Figure 9:**
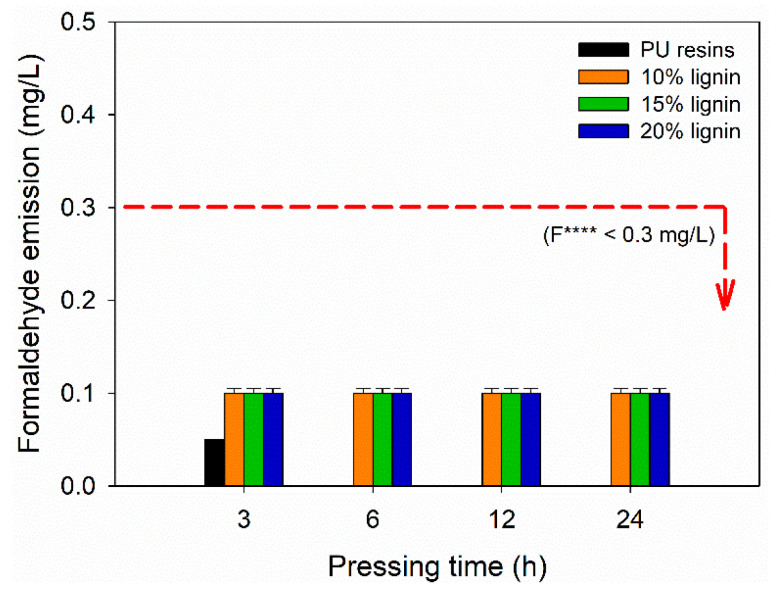
Formaldehyde emission of plywood bonded with PVOH–lignin–hexamine-based adhesives at different lignin contents and pressing times.

The FE of plywood bonded with PVOH–lignin–hexamine-based adhesives was evaluated according to JAS No. 233:2003 [43]. Figure 9 display the FE values of plywood as a function of lignin content and different pressing times. The formulation of PVOH–lignin–hexamine-based adhesives did not use formaldehyde; therefore, the resulting plywood exhibited close-to-zero formaldehyde emission. Hexamine is derived from formaldehyde, but decomposition of hexamine into formaldehyde occurs at elevated temperatures [35]. Since the obtained plywood was prepared using cold pressing, the FE value was lower compared with the formaldehyde-based adhesives. An average of 0.1 mg/L of FE value from plywood bonded with PVOH–lignin–hexamine-based adhesives and 0.05 mg/L of FE for the control were measured according to the desiccator method. No remarkable effect was observed for FE values at different contents of lignin and different pressing times. The resulting FE could be the biogenic formaldehyde originating from wood, which was in agreement with the published works [71,72,73,74,75]. It is well acknowledged that wood contains biogenic formaldehyde from lignin. The FE values in this study were lower than 0.3 mg/L of the F **** according to the JAS No. 233:2003 [43].

## 4. Conclusions

Cold-setting plywood adhesive was developed based on PVOH–lignin–hexamine blends. The influence of lignin content (10%, 15%, and 20%) and cold-pressing time (3, 6, 12, and 24 h) on the cohesion, adhesion, and formaldehyde emission of plywood were investigated by physical, chemical, thermal, and mechanical analyses. This study showed that the increased lignin content lowered the solids content and average viscosity of the adhesive. Markedly, the cohesion strength of the adhesive formulation comprising 10% lignin addition, around 831 Pa, was greater than those of 15% and 20% lignin content. The results obtained for the thermo-mechanical properties of PVOH–lignin–hexamine-based adhesives showed that the addition of 15% lignin content in the adhesive mixture resulted in the highest storage modulus values of about 450 GPa at 48 °C. To note, the addition of 10% and 15% lignin content in the adhesive resulted in better cohesion strength compared to the adhesive containing 20% lignin content. This study revealed that the addition of 20% lignin in the adhesive and using cold-pressing times of 12 h and 24 h could produce a plywood with a tested adhesive that met the Japanese standard strength requirements, with a TSS value of 0.95 MPa, MOR values ranging from 30–36 MPa, MOE values varying from 3800–4000 MPa, and FE of 0.1 mg/L (<0.3 mg/L of F ****). This study showed the possibility of fabricating eco-friendly plywood panels bonded with PVOH–lignin–hexamine-based adhesive using the cold-pressing process as an alternative to conventional plywood.

## Figures and Tables

**Table 1:** Formulation of PVOH–lignin–hexamine-based adhesive at different lignin contents.

Lignin Content (%)	Chemicals			Total
	PVOH *	Lignin **	Hexamine ***	
10	100.0	15.0	15.0	130.0
15	100.0	22.5	15.0	137.5
20	100.0	30.0	15.0	145.0

* PVOH was the base of adhesive, ** Lignin in acetone (1:10) was added based on the solid content of PVOH, *** Hexamine was added based on the solid content of PVOH.

**Table 2:** Fabrication of plywood bonded with PVOH–lignin–hexamine-based adhesive at different lignin contents.

Lignin Content (%)	Pressing Time (h)				Number of Plywood
	3	6	12	24	
Control *	3	-	-	-	3
10	3	3	3	3	12
15	3	3	3	3	12
20	3	3	3	3	12

* control was PU resins.

**Table 3:** Properties of lignin from black liquor.

Parameters	Value	References
Solids content of black liquor (%)	76.8 ± 0.64	65.0–85.0 [56,57]
Yield of lignin (%)	35.9 ± 1.81	35.0–46.0 [49,55]
MC of lignin(%)	5.1 ± 0.71	5.0–8.0 [49,55]
Ash content of lignin (%)	0.3 ± 0.19	8.3–19.2 [49,55]
AIL (%)	82.5 ± 0.96	53.1 [49,55]
ASL (%)	12.8 ± 0.67	7.3 [49,55]
Purity of lignin (%)	95.3 ± 0.61	60.4 [49,55]

**Table 4:** Basic properties of PVOH–lignin–hexamine-based adhesives.

Lignin Content (%)	Solids Content (%)	Viscosity (mPa·s)	Gelation Time (min)
Control *	96.52 ± 0.25	2056.5 ± 50.31	187.5 ± 2.0
10	25.96 ± 0.24	1894.6 ± 165.2	nd **
15	22.98 ± 0.27	1309.9 ± 82.08	nd
20	19.31 ± 0.31	968.5 ± 65.59	nd

* control was PU resins, ** not detected by the gel time meter at 25 °C (max 999 min).

**Table 5:** Physical properties of plywood bonded with PVOH–lignin–hexamine-based adhesives at different lignin contents and pressing times.

Lignin Content (%)	Pressing Time (h)	Density (g/cm^3^)	Moisture Content (%) **	Delamination (%) ***
Control *	3	0.42 ± 0.02	5.34 ± 0.09	0.0 ± 0.0
10	3	0.42 ± 0.01	8.95 ± 0.02	100.0 ± 0.0
	6	0.42 ± 0.03	9.37 ± 0.07	100.0 ± 0.0
	12	0.42 ± 0.04	9.69 ± 0.05	100.0 ± 0.0
	24	0.42 ± 0.02	12.05 ± 0.06	100.0 ± 0.0
15	3	0.42 ± 0.02	8.98 ± 0.08	100.0 ± 0.0
	6	0.42 ± 0.05	9.37 ± 0.07	100.0 ± 0.0
	12	0.42 ± 0.04	9.71 ± 0.04	100.0 ± 0.0
	24	0.42 ± 0.07	12.82 ± 0.07	100.0 ± 0.0
20	3	0.42 ± 0.06	9.27 ± 0.03	100.0 ± 0.0
	6	0.42 ± 0.07	9.37 ± 0.03	100.0 ± 0.0
	12	0.42 ± 0.08	9.89 ± 0.04	100.0 ± 0.0
	24	0.42 ± 0.02	12.98 ± 0.09	100.0 ± 0.0

* control adhesive was PU resins, ** maximum moisture content was 14%, *** maximum non-delaminated part was less than 50 mm (delamination < 33.3%).

**Table 6:** Statistical analysis of mechanical properties of plywood bonded with PVOH–lignin–hexamine-based adhesives at different lignin contents and pressing times.

Properties	Variable	Mean Square	F-Value	*p*-Value
TSS	Lignin content	0.347	10.601	0.001 *
	Pressing time	0.116	2.212	0.014
	Interaction	0.155	12.514	0.001
MOR	Lignin content	913.573	24.275	0.001
	Pressing time	78.337	0.717	0.048
	Interaction	267.256	8.166	0.001
MOE	Lignin content	8,217,578.762	18.188	0.001
	Pressing time	1,721,046.403	1.706	0.018
	Interaction	3,054,108.955	20.805	0.001

* The variable with *p*-value lower than α = 0.05 is significantly influence the mechanical properties of plywood.

## Data Availability

Not applicable.
